# Racial and Ethnic Disparities in Initiation of Direct Oral Anticoagulants Among Medicare Beneficiaries

**DOI:** 10.1001/jamanetworkopen.2024.9465

**Published:** 2024-05-06

**Authors:** Kamika R. Reynolds, Farzin Khosrow-Khavar, Chintan V. Dave

**Affiliations:** 1 Center for Pharmacoepidemiology and Treatment Science, Institute for Health, Health Care Policy and Aging Research, Rutgers University, New Brunswick, New Jersey; 2 Department of Biostatistics and Epidemiology, Rutgers School of Public Health, Piscataway, New Jersey; 3 Rutgers Cancer Institute of New Jersey, New Brunswick; 4 Department of Pharmacy Practice and Administration, Ernest Mario School of Pharmacy, Rutgers University, Piscataway, New Jersey

## Abstract

**Importance:**

The influence of race and ethnicity on initiation of direct oral anticoagulants (DOACs) is relatively understudied in Medicare data.

**Objective:**

To investigate disparities in the initiation of DOACs compared with warfarin by race, ethnicity, and social vulnerability.

**Design, Setting, and Participants:**

This retrospective cohort study used a 50% sample of Medicare fee-for-service data from January 1, 2010, to December 31, 2019 (mean patient enrollment duration, 7.7 years). Analysis took place between January 2023 and February 2024. A cohort of older adults (aged ≥65 years) with atrial fibrillation who newly initiated warfarin or DOACs (dabigatran, rivaroxaban, apixaban, and edoxaban) was identified.

**Exposure:**

Patients were classified as non-Hispanic White, non-Hispanic Black, and Hispanic.

**Main Outcomes and Measures:**

The likelihood of starting use of DOACs compared with warfarin was modeled, adjusting for race, ethnicity, age, sex, county-level social vulnerability, and other clinical factors.

**Results:**

Among 950 698 anticoagulation initiations, consisting of 680 974 DOAC users and 269 724 warfarin users (mean [SD] age, 78.5 [7.6] years; 52.6% female), 5.2% were Black, 4.3% were Hispanic, and 86.7% were White. During the 10-year study period, DOAC use increased for all demographic groups. After adjustment, compared with White patients, Black patients were 23% less likely (adjusted odds ratio [AOR, 0.77; 95% CI, 0.75-0.79) and Hispanic patients were 13% less likely (AOR, 0.87; 95% CI, 0.85-0.89) to initiate DOAC use. Disparities in DOAC initiation were greatest among Black patients in the earlier years but attenuated during the study period. For instance, in 2010, the OR of Black patients initiating DOACs was 0.54 (95% CI, 0.50-0.57), attenuating linearly over time to 0.69 by 2013 (95% CI, 0.65-0.74) and 0.83 (95% CI, 0.78-0.89) by 2017. By 2019, these differences became nonsignificant (OR, 1.08; 95% CI, 0.99-1.18).

**Conclusions and Relevance:**

In this cohort study of Medicare patients with atrial fibrillation, Black and Hispanic patients were less likely to initiate DOACs for atrial fibrillation, although these differences diminished over time. Identifying the factors behind these early disparities is crucial for ensuring equitable access to novel therapies as they emerge for Black and Hispanic populations.

## Key Points

**Question:**

How are race, ethnicity, and social vulnerability associated with the new initiation of direct oral anticoagulants (DOACs) among older US adults (aged ≥65 years) with atrial fibrillation?

**Findings:**

In this cohort study of 950 698 anticoagulation initiation episodes from 2010 to 2019, after adjustment, Black and Hispanic patients were 23% and 13% less likely to initiate DOACs, respectively. Disparities in DOAC initiation were greatest among Black patients in earlier years but attenuated during the study period and dissipated entirely by 2019.

**Meaning:**

This study highlights the evolution of atrial fibrillation management, underscoring historical imbalances that have shown signs of abatement.

## Teaser

This cohort study investigates potential disparities by race, ethnicity, and social vulnerability in the initiation of direct oral anticoagulants among Medicare beneficiaries in the US.

## Introduction

Atrial fibrillation (AF) is a frequently occurring cardiac arrhythmia characterized by substantial morbidity and mortality.^1^ In the US, the incidence and prevalence of AF are lower in minority populations compared with White populations; however, underrepresented minorities are less likely to be diagnosed with and more likely to be undertreated for AF.^2,3^ Such disparities in detection and treatment may also play a role in the more severe thrombotic events and higher mortality experienced by minority patients with AF compared with their White counterparts.^3^

Thromboembolism is the most severe consequence of AF; however, oral anticoagulants can reduce its incidence by 70% regardless of baseline risk.^4^ Warfarin has been the anticoagulant of choice for decades,^5^ but several challenges, including dietary restrictions, drug interactions, and the requirement for frequent clinical monitoring, accompany its use.^6^ The landscape of AF treatment has shifted since 2010 with the approval of the following direct oral anticoagulants (DOACs): dabigatran (2010), rivaroxaban (2011), apixaban (2012), and edoxaban (2015).^7^ Clinical trials have shown that DOACs are just as effective as warfarin for the prevention of stroke, myocardial infarction, and all-cause mortality among patients with AF.^8^ Moreover, unlike warfarin, DOACs have a predictable anticoagulant effect, eliminate the need for routine clinical monitoring, and pose fewer dietary restrictions and drug interactions.^9^

Consequently, prevailing clinical guidelines now advocate for the use of DOACs over warfarin for use in patients with AF.^10,11^ Prior studies using US health care claims data have demonstrated consistent augmentation in DOAC prescription trends, with DOAC prescriptions outpacing those of warfarin in recent years.^12^ Despite this trend toward increasing DOAC use, existing research has highlighted treatment disparities among racial and ethnic groups.^2,13,14,15^ However, the exploration of how these novel anticoagulants are adopted across diverse racial and ethnic populations, especially in recent years, factoring in social determinants of health, stroke risk levels, and the changes in treatment disparities over time, remains relatively understudied in Medicare data. Given that older adults have the highest prevalence of AF of any age group,^16^ it becomes especially imperative to examine this population. Therefore, this study aimed to explore potential disparities in DOAC uptake between non-Hispanic Black and Hispanic patients relative to non-Hispanic White patients using Medicare data from 2010 to 2019. It also aimed to assess how these disparities manifest and evolve over time.

## Methods

The Rutgers University Institutional Review Board approved this cohort study, and the requirement for informed consent was waived. We followed the Strengthening the Reporting of Observational Studies in Epidemiology (STROBE) reporting guideline.^17^

### Data Sources and Study Population

Study participants were drawn from Medicare insurance claims, a US federal program that provides health care to US citizens 65 years or older. More specifically, we used a 50% random sample of Medicare fee-for-service beneficiaries enrolled in Part D from January 1, 2010, to December 31, 2019 (mean enrollment duration, 7.7 years). Medicare provides patient-level information on pharmacy and health care enrollment; sociodemographic variables, including date of birth, sex, and race and ethnicity, as well as information on inpatient and outpatient services (*International Classification of Diseases, Ninth Revision* [*ICD-9*], *International Statistical Classification of Diseases and Related Health Problems, Tenth Revision* [*ICD-10*], and *Current Procedural Terminology*, Fourth Edition codes); and outpatient pharmacy dispensing (drug name, date of dispensing, and days’ supply).

Within the data, we identified patients with 1 inpatient or outpatient diagnosis code of AF or flutter (*ICD-9* code 427.3x or *ICD-10* code I48.xx)^18^ who newly initiated use of the following anticoagulants: dabigatran, rivaroxaban, apixaban, edoxaban, or warfarin. Treatment initiation was defined as the nonuse of the study medication for a minimum of the baseline period (ie, 365 days before treatment initiation) but extending to the entirety of the available lookback period. Cohort membership was further restricted to patients without evidence of valvular heart disease, venous thromboembolism, cancer, or hip or knee surgery and patients who had continuous data for at least 1 year before their index date (ie, date of treatment initiation). Patients were allowed to contribute more than 1 episode of treatment initiation as long as the eligibility criteria were met at the time of initiation.

### Patient Characteristics

To describe the study population, we examined additional patient characteristics, such as age, sex, select comorbid conditions (eg, stroke or heart failure), and select prescription medications (eg, antihypertensives or statins). All baseline conditions, including the eligibility criteria, were assessed during the baseline period (365 days before the index date).

Medicare race and ethnicity information is available from the Social Security Administration enrollment data. The Research Triangle Institute (RTI) modified race variable was used to classify study participants into the following categories of race and ethnicity: non-Hispanic Black (Black), Hispanic, and non-Hispanic White (White) (other races were excluded because of small sample sizes). An algorithm was developed to impute race because Hispanic patients are undercounted in the Medicare enrollment database race variable.^19^ When compared with the gold standard of self-reported race, the positive predictive value for identifying Black and White Medicare beneficiaries exceeds 95% and ranges from 85% to 90% for Hispanic patients when using the RTI race and ethnicity variable, with specificity rates surpassing 90% for all groups.^20^ American Indian and Alaska Native individuals, Asian American and Pacific Islander individuals, and individuals with other or missing race information were collapsed into an other race category, accounting for less than 4% of our study population. Information on race and ethnicity was missing for 6896 (0.7%) of the cohort.

Using the patient county of residence at the time of anticoagulant initiation, we linked patient records to the Social Vulnerability Index (SVI) data for 2010, 2014, 2016, and 2018.^21^ Briefly, the SVI quantifies the relative vulnerability of each US county, ranking them from the least to most vulnerable, with higher scores corresponding to greater vulnerability using 4 themes corresponding to socioeconomic status, household characteristics, racial and ethnic minority status, and housing type and transportation. Each of the 4 SVI themes and the overall SVI score were categorized into quartiles for our analysis.

### Statistical Analysis

All analyses were performed between January 2023 and February 2024 using SAS software, version 9.4 (SAS Institute Inc). The period from 2010 to 2019 was segmented into 10 calendar year intervals, and each episode of treatment initiation was assigned to 1 of these intervals according to the index date. We used all available lookback data for each patient to determine the first dispensing of a particular medication of interest. For each calendar year, we described the proportion of initiations attributable to either a DOAC or warfarin (numerator) over all anticoagulant initiations (denominator). We also described the overall trends in drug initiation patterns during the 10-year period. Logistic regressions estimated the odds of initiating DOACs (ie, response variable), with race and ethnicity as the primary independent variable of interest. More specifically, we examined the odds of initiating DOACs for Black and Hispanic recipients compared with their White counterparts. We estimated 4 models: (1) an unadjusted model accounting solely for race and ethnicity; (2) a minimally adjusted model adjusting for age and sex only; (3) a partially adjusted model adjusting for age, sex, and SVI themes; and (4) a fully adjusted model adjusting for age, sex, SVI themes, comorbid conditions, and prescription medications described in the Table. All analyses accounted for potential clustering at the patient level, and all hypothesis tests were conducted at a 2-sided significance level of *P* < .05.

**Table.** Baseline Characteristics of US Patients Prescribed Warfarin and DOACs From 2010 to 2019^a^

Characteristic	Warfarin (n = 269 724)	DOAC (n = 680 974)
Age, mean (SD), y	78.4 (7.6)	78.6 (7.6)
Prior anticoagulant use^b^	26 632 (9.9)	151 971 (22.3)
Sex		
Female	144 700 (53.7)	355 511 (52.2)
Male	125 024 (46.4)	325 463 (47.8)
Race and ethnicity		
Black	16 778 (6.2)	32 991 (4.8)
Hispanic	11 965 (4.4)	28 739 (4.2)
White	232 426 (86.2)	591 598 (86.9)
Other^c^	8555 (3.2)	27 646 (4.1)
Year		
2010-2012	124 086 (46.0)	74 289 (10.9)
2013-2016	101 310 (37.6)	278 413 (40.9)
2017-2019	44 328 (16.4)	328 272 (48.2)
CHA_2_DS_2_-VASc score, mean (SD)	5.4 (1.8)	5.1 (1.8)
Comorbid conditions		
CKD	54 749 (20.3)	120 126 (17.6)
Stroke	75 265 (27.9)	166 532 (24.5)
MI	28 251 (10.5)	48 320 (7.1)
Heart failure	118 267 (43.9)	251 375 (36.9)
Hypertension	247 911 (91.9)	621 682 (91.3)
COPD	82 710 (30.7)	178 554 (26.2)
Prescription medications		
Antiplatelet	44 899 (16.7)	93 688 (13.8)
Antidiabetic	74 646 (27.7)	170 780 (25.1)
ARB	60 832 (22.6)	183 329 (26.9)
ACEi	93 816 (34.8)	215 088 (31.6)
β-Blocker	139 272 (51.6)	376 071 (55.2)
CCB	77 381 (28.7)	187 278 (27.5)
Nondihydropyridine CCB	39 331 (14.6)	114 703 (16.8)
Thiazide diuretic	55 752 (20.7)	136 638 (20.1)
Loop diuretic	90 618 (33.6)	221 198 (32.5)
Antiarrhythmic	30 623 (11.4)	108 272 (15.9)
Statin	150 237 (55.7)	412 556 (60.6)
Social Vulnerability Index themes, mean (SD) percentiles^d^		
Socioeconomic status	0.43 (0.26)	0.43 (0.25)
Household composition and disability	0.38 (0.27)	0.36 (0.27)
Minority status and language	0.68 (0.27)	0.71 (0.25)
Housing type and transportation	0.59 (0.26)	0.60 (0.26)
Overall	0.51 (0.26)	0.52 (0.25)

Abbreviations: ACEi, angiotensin-converting enzyme inhibitors; ARB, angiotensin receptor blocker; CCB, calcium channel blocker; CHA_2_DS_2_-VASc, congestive heart failure, hypertension, age of 75 years or older (doubled), diabetes, stroke (doubled), vascular disease, age 65 to 74 years, and sex category (female); CKD, chronic kidney disease; COPD, chronic obstructive pulmonary disease; DOAC, direct oral anticoagulant; MI, myocardial infarction.

^a^ Data are presented as number (percentage) of patients unless otherwise indicated.

^b^ Prior warfarin use for DOAC users and prior DOAC use for warfarin users.

^c^ The other race category comprised American Indian or Alaska Native individuals, Asian American or Pacific Islander individuals, and other racial and ethnic groups not otherwise defined.

^d^ Socioeconomic status measures income, poverty, employment, and educational level; household composition and disability measures age, single parenting, and disability; minority status and language measures race and ethnicity; and housing and transportation measures housing structure, crowding, and vehicle access.

Our decision to estimate 4 separate models was driven by distinct rationales. The first, an unadjusted model, focuses exclusively on race and ethnicity to provide a foundational understanding of how the initiation of novel anticoagulants varies across racial and ethnic groups without considering other variables. The second model incorporates age and sex, addressing their potential confounding influences on anticoagulant use. The third model further integrated social determinants of health information, enabling assessment of any additional attenuation associated with these factors. Finally, the fully adjusted model (ie, primary analysis) also adjusted for patient characteristics and variables, considering differences in pertinent clinical presentation that may impact receipt of DOACs. Moreover, for a temporal analysis, we computed both unadjusted and fully adjusted models for each year within the study timeframe, facilitating a detailed examination of trends and disparities in DOAC initiation over time.

We also conducted 3 sensitivity analyses. First, instead of examining DOACs as a drug class, we estimated multinomial regression models to model the odds of using individual agents within the class (ie, dabigatran, apixaban, rivaroxaban, or edoxaban) relative to warfarin for Black and Hispanic patients compared with White patients. For this analysis, the study cohort was restricted to treatment-naive patients, defined as those who had not previously used an anticoagulant at any point before their first use. Second, we evaluated disparities in the initiation of these medications by race and ethnicity within the highest and lowest quartiles of the SVI to discern whether differences in medication initiation were exacerbated or mitigated among patients across the extremes of SVI strata. Third, we investigated disparities in medication initiation across different levels of stroke risk, as assessed by CHA_2_DS_2_-VASc (congestive heart failure, hypertension, age ≥75 years [doubled], diabetes, stroke [doubled], vascular disease, age 65 to 74 years, and sex category [female]) scores. We analyzed patients with scores above and below the median and those within the highest risk quartile.

## Results

After the inclusion and exclusion criteria were applied, we identified 269 724 new users of warfarin and 680 972 new users of DOACs (mean [SD] age, 78.5 [7.6] years; 52.6% female and 47.4% male) (eFigure 1 in Supplement 1). Black patients comprised 5.2% of the population; Hispanic, 4.3%; and White, 86.7%. The baseline characteristics of patients initiating warfarin and DOACs were similar with respect to age, biological sex, and other comorbid conditions, such as hypertension; however, higher rates of certain conditions, such as chronic kidney disease, stroke, myocardial infarction, and heart failure, were noted among warfarin users (Table; see eTable 1 in Supplement 1 for characteristics of patients initiating use of individual DOACs). Warfarin initiation was more common among Black patients than in White or Hispanic patients. The mean (SD) CHA_2_DS_2_-VASc score was higher in warfarin initiators (5.4 [1.80]) than DOAC initiators (5.1 [1.8]). The mean SVI percentiles overall and by themes did not vary appreciably between the 2 groups.

### Medication Initiation Patterns Overall and by Race and Ethnicity

**Figure 1.**
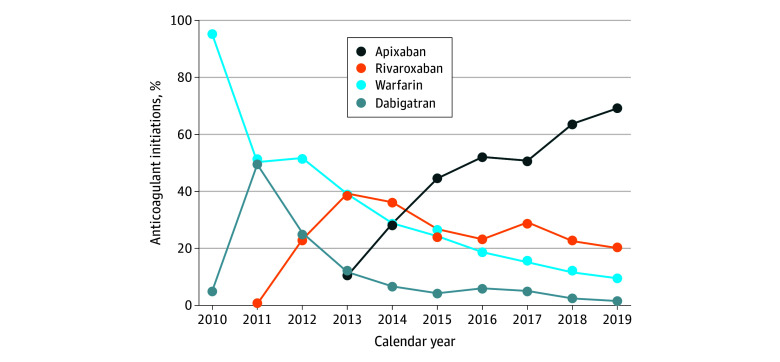
Uptake of Anticoagulants Among US Patients Prescribed Warfarin and Direct Oral Anticoagulants From 2010 to 2019

Among the overall study population and during the study period, warfarin initiations as a proportion of overall anticoagulant initiations decreased significantly by 85.7 percentage points from 95.1% in 2010 to 9.4% in 2019 (Figure 1). This decrease was initially driven by the introduction of dabigatran in 2011 and, to a smaller extent, rivaroxaban in 2013. However, the most substantial contributor to the reduction in warfarin initiations was apixaban, which experienced an increase of 58.8 percentage points from 10.3% of initiations in 2013 to 69.1% in 2019. Edoxaban use remained negligible during the study period.

**Figure 2.**
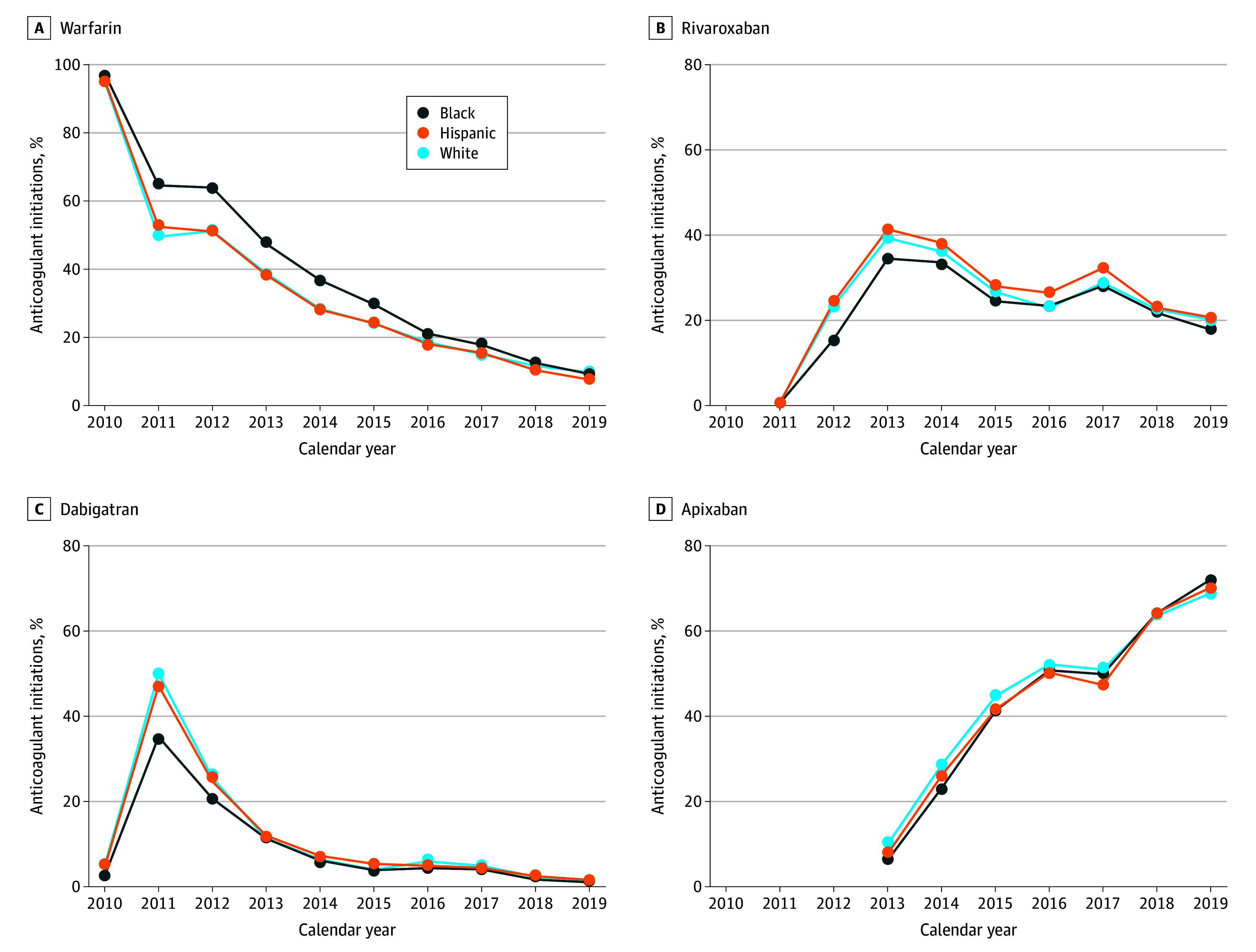
Uptake of Warfarin and Direct Oral Anticoagulants Among US White, Black, and Hispanic Populations From 2010 to 2019

Although DOAC uptake increased for all racial and ethnic groups during the study period, there was evidence of disproportionately lower uptake of DOACs among Black compared with White and Hispanic patients (Figure 2; eTable 2 in Supplement 1). In the beginning of the study period in 2010, 95.0% of White, 95.4% of Hispanic, and 97.4% of Black patients initiated warfarin. Just 1 year later in 2011, warfarin initiations decreased to 49.5%, 52.4%, and 64.5% among White, Hispanic, and Black patients, respectively. These differences persisted during the ensuing years but diminished in severity, dissipating around 2016, when 18.5% of White, 21.0% of Black, and 18.0% of Hispanic patients initiated warfarin. By the end of the study period in 2019, the differences in initiations were mostly eliminated, with warfarin comprising 9.6%, 7.5%, and 9.2% for White, Black, and Hispanic patients, respectively. These patterns were reflected in individual DOACs as well.

### Odds of DOAC Initiation by Race and Ethnicity Overall and by Calendar Year

**Figure 3.**
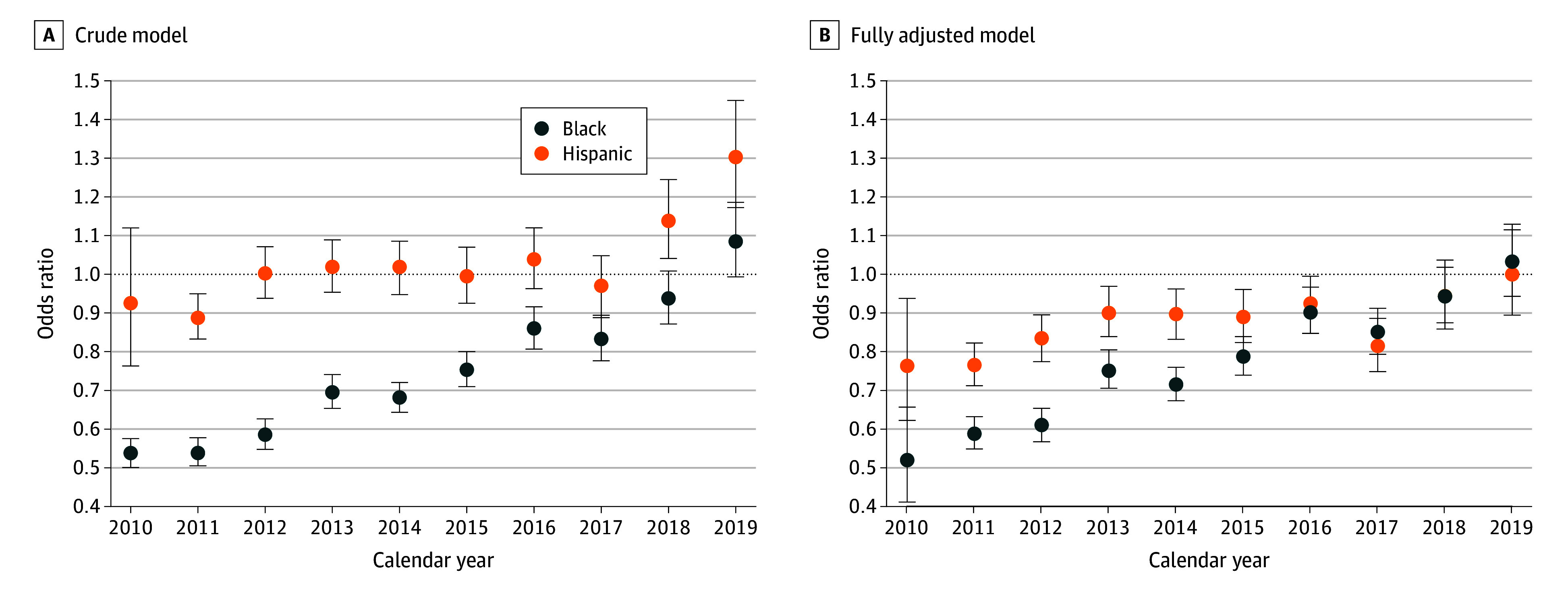
Odds Ratios for Direct Oral Anticoagulant Initiation in Black and Hispanic Patients vs White Patients From 2010 to 2019 Error bars indicate 95% CIs.

In the unadjusted logistic regression analyses, we observed that Black patients had a reduced odds of DOAC initiation throughout most of the study period compared with White patients (Figure 3A and eTable 6 in Supplement 1). For instance, in 2010, the odds ratio (OR) of Black patients initiating a DOAC was 0.54 (95% CI, 0.50-0.57), attenuating linearly over time to 0.69 by 2013 (95% CI, 0.65-0.74) and 0.83 (95% CI, 0.78-0.89) by 2017. By 2019, these differences became nonsignificant (OR, 1.08; 95% CI, 0.99-1.18). Hispanic patients compared with White patients had similar or increased odds of DOAC initiation for most of the study period.

In the minimally adjusted model (ie, age and sex only) and throughout the study period, Black and Hispanic compared with White patients were less likely (Black: adjusted OR [AOR], 0.78; 95% CI, 0.76-0.79; Hispanic: AOR, 0.95; 95% CI, 0.93-0.97) to initiate DOACs (eTable 3 in Supplement 1). These disparities in uptake became more pronounced when the models were further adjusted for SVI themes (Black: AOR, 0.66; 95% CI, 0.64-0.67; Hispanic: AOR, 0.79; 95% CI, 0.77-0.81).

In the fully adjusted model, when baseline comorbid conditions, prescription use, and the SVI themes were all accounted for, the trends observed among Black patients were still present, with Black race and Hispanic ethnicity associated with lower odds of DOAC initiation (Black: AOR, 0.77; 95% CI, 0.75-0.79; Hispanic: AOR, 0.87; 95% CI, 0.85-0.89) compared with their White counterparts. The trends were also largely similar between the crude and fully adjusted models (Figure 3B), with some exceptions. For Hispanic patients, the adjusted model between 2010 and 2012 had larger reductions in the odds of DOAC initiation compared with White patients. Furthermore, the increase in the odds of DOAC initiation for Hispanic patients were attenuated entirely after adjustment.

### Sensitivity Analyses

Study results examining the odds of initiation of dabigatran, apixaban, rivaroxaban, or edoxaban relative to warfarin for Black and Hispanic patients compared with White patients were consistent with the primary analysis (eTable 4 in Supplement 1). When the analyses were limited to treatment-naive patients newly initiating use of an anticoagulant for the first time, results were similar to the primary findings (eTable 5 in Supplement 1).

**Figure 4.**
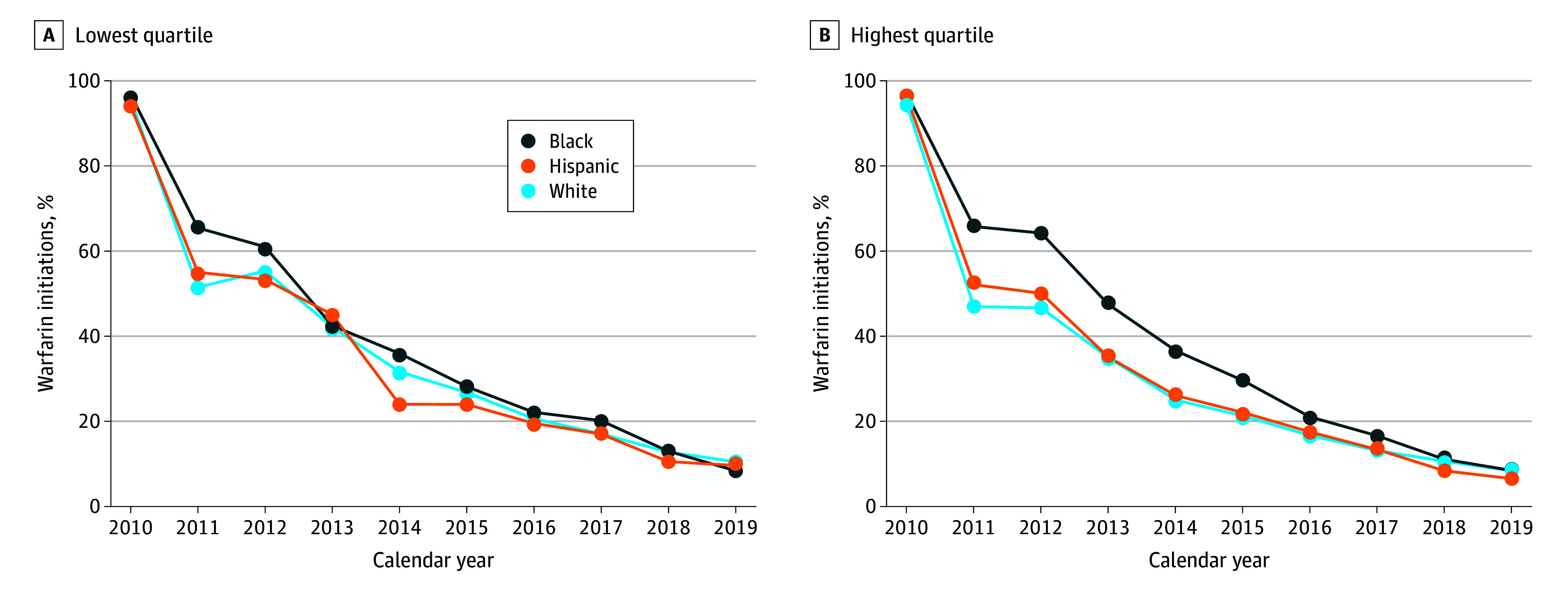
Warfarin Uptake Among White, Black, and Hispanic US Patients in the Lowest and Highest Quartiles of the Overall Social Vulnerability Index Theme From 2010 to 2019

In the analysis examining differences in warfarin uptake by race and ethnicity across the lowest and highest SVI quartiles, the disparities among Black and White patients were attenuated by 2013 in the lowest vulnerability quartile compared with 2018 in the highest vulnerability quartile (Figure 4 and eFigure 2 in Supplement 1). Conversely, among patients in the highest vulnerability quartile, the magnitude in disparities of initiation of warfarin was more pronounced for Black vs White patients compared with the primary analysis but dissipated by the end of the study period in 2019.

In our analysis examining the association between DOAC uptake and CHA_2_DS_2_-VASc scores, we found that Black patients had a slower rate of DOAC initiation compared with White and Hispanic patients across all CHA_2_DS_2_-VASc categories during the early years of the study (eFigure 3 in Supplement 1). These disparities diminished during the study period. However, initially, the disparity in DOAC uptake among Black patients was less pronounced for those with lower CHA_2_DS_2_-VASc scores and increasing for those within the highest score quartile.

## Discussion

The presence of racial and ethnic disparities in DOAC uptake has been hypothesized yet remains relatively understudied within Medicare data, particularly factoring in social determinants of health, stroke risk stratification, and the changing landscape of treatment disparities over time. In response to this knowledge gap, we used Medicare data from 2010 to 2019 to investigate trends and potential inequities in DOAC use across race, ethnicity, and social vulnerability. There were several notable insights from our analysis. In 2010, warfarin was the most commonly initiated anticoagulant, comprising more than 90% of anticoagulants initiated in Medicare. However, the introduction of DOACs resulted in a steep decrease in warfarin use across all patient subgroups. Interestingly, discernible disparities manifested within this waning trend of warfarin use, with Black and Hispanic patients exhibiting a 23% and 13% lower likelihood, respectively, of DOAC use even after adjusting for various pertinent factors, including social vulnerability. These disparities in use were most pronounced in early study years, but their magnitude appeared to ameliorate during the study period, with no discernable disparities during the final year of the study.

Our analysis revealed a pronounced disparity in DOAC initiation between Black and White patients, particularly noticeable among those with the highest CHA_2_DS_2_-VASc scores in the early study years. The observed trend, in which the most significant disparities occurred among the highest-risk patients in the early study years, contradicts the anticipated model of equitable access and treatment prioritization based on patient risk profiles, underscoring a critical area for intervention for novel therapies as they emerge.

In the context of prevailing guidelines recommending DOACs for AF, our investigation uncovers a distinctive pattern of delayed DOAC initiation among Black and Hispanic patients compared with their White counterparts, especially in the earlier years and among Black Medicare beneficiaries. Notably, these inequities in use persisted even after adjustments for clinical characteristics and county-level vulnerability, suggesting the influence of additional underlying factors. Although our study cannot determine the exact reason for this difference in uptake, it may be attributable to several factors. First, one possible reason is the cost disparity between warfarin, which is a generic medication, and branded DOACs. The higher costs associated with DOACs could disproportionately impact Black and Hispanic patients, as underscored by previous research indicating a preference for generic drugs among minority patients without low-income subsidies.^22,23^ Second, clinician prescribing preferences pertaining to anticoagulants may also have contributed. For instance, a previous analysis of Medicare physicians from 2013 to 2018 revealed a higher rate of DOAC initiation among cardiologists.^24^ Given the reduced likelihood of Black and Hispanic patients being under the care of specialists, this aspect could have contributed to the disparity in DOAC use in the initial study years.^13^ Third, implicit biases present in medical decision-making processes, varying levels of patient health literacy, and disparate perceptions of the health care system may also have collectively contributed to the observed disparities in DOAC adoption.^2,25^

Although the study cannot attribute the increase in uptake of DOACs among Black and Hispanic patients in the later study years to any particular cause, several factors may have contributed to this trend. These factors include improving adherence to AF guidelines recommending DOACs over warfarin, increased clinician familiarity with DOACs, and increasing awareness of fewer dietary restrictions and reduced testing requirements among all patients. Furthermore, an increased focus on health equity among health care professionals and systemic improvements in health care delivery may have also facilitated the observed shift toward more equitable DOAC use across racial and ethnic groups.^26^

Our study findings are generally consistent with the available literature on the general trends in DOAC use in the US and globally.^12,14,27,28,29,30^ A Medicare study of older data from 2012 to 2014 found that Black patients had 25% lower odds of initiating DOACs compared with White patients, whereas Hispanic patients exhibited similar odds.^14^ Another investigation of 12 417 patients with AF who were 21 years or older in the Outcomes Registry for Better Informed Treatment of Atrial Fibrillation II registry from 2013 to 2016 found that Black patients were 40% less likely to use DOACs compared with White patients.^13^ However, adjustment for socioeconomic and other factors attenuated the magnitude of the association for Black patients to a 27% reduction in likelihood.^13^ Another study examining patients with AF who were older than 18 years between 2011 and 2017 with a CHA_2_DS_2_-VASc score of 2 or higher revealed that Black and Hispanic patients had 28% and 47% lower odds of DOAC use, respectively.^31^ Our findings correspond with the prior study’s conclusions for Black patients but diverged for Hispanic patients. This divergence could be attributed to either different age groups examined between the 2 studies or our study’s use of Medicare data. Future research might delve into whether such disparities persist among younger Hispanic patients compared with their older counterparts.

### Strengths and Limitations

Several notable strengths characterize this study. It leverages an extensive and highly representative data set, encompassing a geographically and racially diverse sample of older US Medicare beneficiaries 65 years or older with AF. Significantly, the research also delves into adjusted secular trends, shedding light on both the initial years of pronounced disparities followed by an encouraging attenuation of these disparities. Furthermore, the application of stepwise adjustments in our logistic regression models offers a nuanced understanding of the factors that mitigate or amplify the existing disparities.

Study findings should be viewed in light of some limitations. First, information on social vulnerability was aggregated on a county level, and individual-level data were thus unavailable. Second, we did not have information on prescriber specialty, which may have contributed to some of the observed disparities, especially in the initial study years. Third, we were unable to account for formulary differences. However, previous research has shown that the percentage of Part D plans covering DOACs increased from 84% to 100% from 2013 to 2017.^32^ Fourth, despite the high positive predictive value, the categorization of race and ethnicity was based on the RTI variable (and derived from Social Security Administration files) rather than the gold standard of self-report. Fifth, our analysis focused on patients with AF who initiated anticoagulant treatment, thereby excluding those who did not commence any form of anticoagulant therapy. This omission overlooks an additional potential manifestation of health care disparity (ie, treatment with an anticoagulant), which also has substantial implications for patient care and treatment outcomes.^2^

## Conclusions

In this cohort study of Medicare patients 65 years or older with AF, we observed lower DOAC initiation rates among Black and Hispanic patients compared with White patients, especially in the earlier study years. Notably, by the conclusion of our research period, these disparities had fully dissipated, indicating a significant shift toward equitable DOAC use among racial and ethnic groups. This study highlights the evolution of management of AF, underscoring historical imbalances that have shown signs of abatement.

## Notes

Supplement 1. **eFigure 1.** CONSORT Flow Diagram **eTable 1.** Baseline Characteristics of US Patients Prescribed Warfarin and Direct-Acting Oral Anticoagulants From 2010 to 2019 **eTable 2.** Proportion of Warfarin Initiations Among White, Black, and Hispanic US Atrial Fibrillation Patients From 2010 to 2019 **eTable 3.** Unadjusted, Minimally, Partially, and Fully Adjusted Logistic Regression Models Examining the Association Between Race/Ethnic Groups and DOAC Initiation Among Medicare Participants 2010-2019 **eTable 4.** Fully Adjusted Multinomial Regression Models Examining the Association Between Race/Ethnic Groups and DOAC Initiation Among Medicare Participants 2010-2019 **eTable 5.** Fully Adjusted Multinomial Regression Models Examining the Association Between Race/Ethnic Groups and DOAC Initiation Among Medicare Participants Who Are New Anticoagulant Users 2010-2019 **eTable 6.** Odds Ratios for Initiation of DOACS When Comparing Black and Hispanic Patients With White Patients From 2010-2019 **eFigure 2.** Warfarin Uptake US Patients in the Lowest and Highest Quartiles of the Overall SVI Theme by Racial and Ethnic Group From 2010 to 2019 **eFigure 3.** Proportion of DOAC Initiations Among White, Black, and Hispanic US Atrial Fibrillation Patients From 2010 to 2019 by CHA_2_DS_2_-VASc Score

Supplement 2.
Data Sharing Statement
