# Supplementary material for: Racial and Ethnic Disparities in Initiation of Direct Oral Anticoagulants Among Medicare Beneficiaries
Source: JAMA Netw Open. 2024 May 6;7(5):e249465. doi: 10.1001/jamanetworkopen.2024.9465 (PMC11074810; doi:10.1001/jamanetworkopen.2024.9465)
Supplement: Supplement 1. — eFigure 1. CONSORT Flow Diagram eTable 1. Baseline Characteristics of US Patients Prescribed Warfarin and Direct-Acting Oral Anticoagulants From 2010 to 2019 eTable 2. Proportion of Warfarin Initiations Among White, Black, and Hispanic US Atrial Fibrillation Patients From 2010 to 2019 eTable 3. Unadjusted, Minimally, Partially, and Fully Adjusted Logistic Regression Models Examining the Association Between Race/Ethnic Groups and DOAC Initiation Among Medicare Participants 2010-2019 eTable 4. Fully Adjusted Multinomial Regression Models Examining the Association Between Race/Ethnic Groups and DOAC Initiation Among Medicare Participants 2010-2019 eTable 5. Fully Adjusted Multinomial Regression Models Examining the Association Between Race/Ethnic Groups and DOAC Initiation Among Medicare Participants Who Are New Anticoagulant Users 2010-2019 eTable 6. Odds Ratios for Initiation of DOACS When Comparing Black and Hispanic Patients With White Patients From 2010-2019 eFigure 2. Warfarin Uptake US Patients in the Lowest and Highest Quartiles of the Overall SVI Theme by Racial and Ethnic Group From 2010 to 2019 eFigure 3. Proportion of DOAC Initiations Among White, Black, and Hispanic US Atrial Fibrillation Patients From 2010 to 2019 by CHA2DS2-VASc Score [file jamanetwopen-e249465-s001.pdf]

Supplementary Online Content

Reynolds KR, Khosrow-Khavar F, Dave CV. Racial and ethnic disparities in initiation of direct oral anticoagulants among Medicare beneficiaries. *JAMA Netw Open*. 2024;7(5):e249465. doi:10.1001/jamanetworkopen.2024.9465

eFigure 1. CONSORT Flow Diagram

eTable 1. Baseline Characteristics of US Patients Prescribed Warfarin and Direct-Acting Oral Anticoagulants From 2010 to 2019

eTable 2. Proportion of Warfarin Initiations Among White, Black, and Hispanic US Atrial Fibrillation Patients From 2010 to 2019

eTable 3. Unadjusted, Minimally, Partially, and Fully Adjusted Logistic Regression Models Examining the Association Between Race/Ethnic Groups and DOAC Initiation Among Medicare Participants 2010-2019

eTable 4. Fully Adjusted Multinomial Regression Models Examining the Association Between Race/Ethnic Groups and DOAC Initiation Among Medicare Participants 2010-2019

eTable 5. Fully Adjusted Multinomial Regression Models Examining the Association Between Race/Ethnic Groups and DOAC Initiation Among Medicare Participants Who Are New Anticoagulant Users 2010-2019

eTable 6. Odds Ratios for Initiation of DOACS When Comparing Black and Hispanic Patients With White Patients From 2010-2019

eFigure 2. Warfarin Uptake US Patients in the Lowest and Highest Quartiles of the Overall SVI Theme by Racial and Ethnic Group From 2010 to 2019

eFigure 3. Proportion of DOAC Initiations Among White, Black, and Hispanic US Atrial Fibrillation Patients From 2010 to 2019 by CHA_2DS_2-VASc Score

This supplementary material has been provided by the authors to give readers additional information about their work.

© 2024 Reynolds KR et al. *JAMA Network Open.*

eFigure 1. CONSORT Flow Diagram

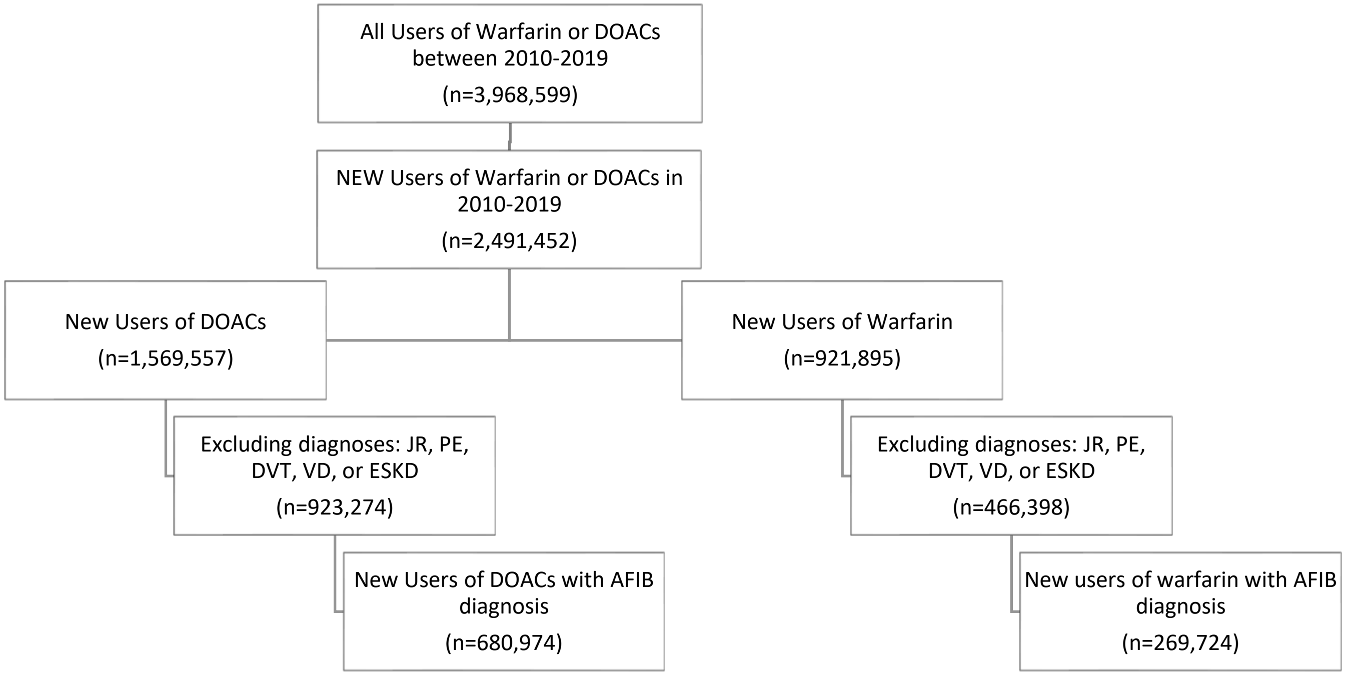

JR, joint replacement; PE, pulmonary embolism; DVT, deep vein thrombosis; VD, valvular disease; ESKD, end stage kidney disease

© 2024 Reynolds KR et al. *JAMA Network Open.*

eTable 1. Baseline Characteristics of US Patients Prescribed Warfarin and Direct-Acting Oral Anticoagulants From 2010 to 2019

Patient Characteristics	Warfarin<br>N= 269724	Apixaban<br>N=364677	Dabigatran<br>N=93915	Edoxaban<br>N=1510	Rivaroxaban<br>N=220872
Age, mean (SD), y	78.4 (7.6)	79.0 (7.7)	78.1 (7.3)	78.8 (7.3)	78.13 (7.5)
Prior Anticoagulant Use[1]	26,632 (9.9)	62,407(17.1)	38,022 (40.5)	280 (17.2)	51,282 (23.2)
Sex					
Female	144,700 (53.7)	191,481 (52.5)	49,646 (52.9)	764 (50.6)	113,620 (51.4)
Race/Ethnicity					
White	232,426 (86.2)	317,157 (87.0)	81,914 (87.2)	1292 (85.6)	191,235 (86.6)
Black	16,778 (6.2)	18,452 (5.1)	3,876 (4.1)	74 (4.9)	10,589 (4.8)
Hispanic	11,965 (4.4)	14,429 (4.0)	4,267 (4.5)	78 (5.2)	9,965 (4.5)
Other	8,555 (3.2)	14,639 (4.0)	3,858 (4.1)	66 (4.4)	9,083 (4.1)
Year					
2010 – 2012	124,086 (46.0)	0	57,701 (61.4)	0	16,588 (7.5)
2013 – 2016	101,310 (37.6)	135,811 (37.2)	25,576 (27.2)	1021 (67.6)	116,005 (52.5)
2017 – 2019	44,328 (16.4)	228,866 (62.8)	10,638 (11.3)	489 (32.4)	88,279 (40.0)
CHA2DS2-VASc, mean (SD)	5.4 (1.8)	5.2 (1.8)	5.1 (1.8)	5.1 (1.8)	5.0 (1.8)
CHA2DS2-VASc, median (IQR)					
Overall	5.0 (4.0-7.0)	5.0 (4.0-6.0)	5.0 (4.0-6.0)	5.0 (4.0-6.0)	5.0 (4.0-6.0)
White	5.0 (4.0-7.0)	5.0 (4.0-6.0)	5.0 (4.0-6.0)	5.0 (4.0-6.0)	5.0 (4.0-6.0)
Black	6.0 (5.0-7.0)	6.0 (5.0-7.0)	6.0 (4.0-7.0)	5.0 (4.0-7.0)	6.0 (4.0-7.0)
Hispanic	6.0 (5.0-7.0)	6.0 (5.0-7.0)	6.0 (4.0-7.0)	6.0 (4.0-7.0)	6.0 (4.0-7.0)
Other	5.0 (4.0-7.0)	5.0 (4.0-6.0)	5.0 (4.0-6.0)	5.0 (4.0-6.0)	5.0 (4.0-6.0)
Comorbid Conditions					
CKD	54,749 (20.3)	77,450 (21.2)	10,640 (11.3)	293 (19.4)	31,743 (14.4)
Stroke	75,265 (27.9)	92,120 (25.3)	23,357 (24.9)	344 (22.8)	50,711 (23.0)
MI	28,251 (10.5)	29,368 (8.1)	4,924 (5.2)	69 (4.6)	13,959 (6.3)
Heart Failure	118,267 (43.9)	139,732 (38.3)	34,762 (37.0)	544 (36.0)	76,337 (34.6)
Hypertension	247,911 (91.9)	334,196 (91.6)	85,744 (91.3)	1379 (91.3)	200,363 (90.7)
COPD	82,710 (30.7)	97,505 (26.7)	24,000 (25.6)	363 (24.0)	56,686 (25.7)
Prescription Medications					
Anti-platelet	44,899 (16.7)	51,322 (14.1)	13,098 (14.0)	180 (11.9)	29,088 (13.2)
Anti-diabetic	74,646 (27.7)	92,087 (25.3)	23,773 (25.3)	365 (24.2)	54,555 (24.7)
ARB	60,832 (22.6)	99,967 (27.4)	24,547 (26.1)	481 (31.9)	58,334 (26.4)
ACEi	93,816 (34.8)	111,158 (30.5)	32,802 (34.9)	419 (27.8)	70,709 (32.0)
Beta Blockers	139,272 (51.6)	201,651 (55.3)	52,640 (56.1)	891 (59.0)	120,889 (54.7)

© 2024 Reynolds KR et al. *JAMA Network Open.*

Patient Characteristics	Warfarin N= 269724	Apixaban N=364677	Dabigatran N=93915	Edoxaban N=1510	Rivaroxaban N=220872
Calcium Channel Blockers	77,381 (28.7)	103,753 (28.5)	24,490 (26.1)	422 (28.0)	58,613 (26.5)
NDHPCCB	39,331 (14.6)	58,440 (16.0)	18,564 (19.8)	272 (18.0)	37,427 (17.0)
Thiazide Diuretics	55,752 (20.7)	70,864 (19.4)	20,608 (21.9)	278 (18.4)	44,888 (20.3)
Loop Diuretics	90,618 (33.6)	118,848 (32.6)	32,849 (35.0)	494 (32.7)	69,007 (31.2)
Antiarrhythmic	30,623 (11.4)	55,648 (15.3)	17,772 (18.9)	360 (23.8)	34,492 (15.6)
Statins	150,237 (55.7)	223,074 (61.2)	56,736 (60.4)	955 (63.3)	131,791 (59.7)
Social Vulnerability Index Themes, Percentiles					
Socioeconomic Status	0.43 ± 0.26	0.43 ± 0.25	0.44 ± 0.26	0.43 ± 0.25	0.43 ± 0.25
Household Composition /Disability	0.38 ± 0.27	0.36 ± 0.27	0.37 ± 0.27	0.33 ± 0.26	0.36 ± 0.27
Minority Status/Language	0.68 ± 0.27	0.71 ± 0.25	0.72 ± 0.26	0.77 ± 0.23	0.72 ± 0.25
Housing Type/Transportation	0.59 ± 0.26	0.60 ± 0.26	0.61 ± 0.26	0.62 ± 0.26	0.60 ± 0.26
Overall	0.51 ± 0.26	0.51 ± 0.25	0.53 ± 0.25	0.53 ± 0.24	0.52 ± 0.25

CKD, Chronic kidney disease; MI, Myocardial infarction; COPD, Chronic obstructive pulmonary disease; ARB, Angiotensin receptor blockers; ACEi, Angiotensin-converting enzyme inhibitors; NDHPCCB, Nondihydropyridine calcium channel blockers; Socioeconomic Status measures income, poverty, employment and education; Household Composition/Disability measures age, single parenting and disability; Minority Status/Language measures race, ethnicity; Housing/ Transportation measures housing structure, crowding, and vehicle access; 1, prior warfarin use for DOAC users and prior DOAC usage for warfarin users

© 2024 Reynolds KR et al. *JAMA Network Open.*

eTable 2. Proportion of Warfarin Initiations Among White, Black, and Hispanic US Atrial Fibrillation Patients From 2010 to 2019

Year	White	Black	Hispanic
2010	95.0	97.4	95.4
2011	49.5	64.5	52.4
2012	51.1	63.9	51.0
2013	38.7	47.4	38.2
2014	28.3	36.7	28.0
2015	23.9	29.6	24.1
2016	18.5	21.0	18.0
2017	15.0	17.7	15.4
2018	11.6	12.4	10.3
2019	9.6	9.2	7.5

© 2024 Reynolds KR et al. *JAMA Network Open.*

eTable 3. Unadjusted, Minimally, Partially, and Fully Adjusted Logistic Regression Models Examining the Association Between Race/Ethnic Groups and DOAC Initiation Among Medicare Participants 2010-2019

a) Unadjusted

**Effect**	**Odds Ratio**	**95% Confidence Interval**	
Black	0.77	0.76	0.79
Hispanic	0.94	0.92	0.97
Other Race	1.27	1.24	1.30

© 2024 Reynolds KR et al. *JAMA Network Open.*

b) Minimally Adjusted

Effect	Odds Ratio	95% Confidence Interval	
Black	0.78	0.76	0.79
Hispanic	0.95	0.93	0.97
Other Race	1.27	1.24	1.30
Age	1.00	1.00	1.00
Female	0.94	0.93	0.95

© 2024 Reynolds KR et al. *JAMA Network Open.*

c) Partially Adjusted

Effect	Odds Ratio	95% Confidence Interval	
Black	0.66	0.64	0.67
Hispanic	0.79	0.77	0.81
Other Race	1.03	1.00	1.06
Age	1.00	1.00	1.00
Female	1.03	1.02	1.04
Socioeconomic Status SVI 2nd quartile	1.10	1.08	1.12
Socioeconomic Status SVI 3rd quartile	1.08	1.05	1.10
Socioeconomic Status SVI 4th quartile	1.21	1.18	1.25
Household Composition /Disability SVI 2nd quartile	0.89	0.87	0.90
Household Composition /Disability SVI 3rd quartile	0.82	0.81	0.84
Household Composition /Disability SVI 4th quartile	0.76	0.74	0.78
Minority Status/Language SVI 2nd quartile	1.26	1.24	1.28
Minority Status/Language SVI 3rd quartile	1.34	1.32	1.36
Minority Status/Language SVI 4th quartile	1.69	1.65	1.72
Housing Type / Transportation SVI 2nd quartile	0.86	0.85	0.87
Housing Type / Transportation SVI 3rd quartile	0.76	0.75	0.78
Housing Type / Transportation SVI 4th quartile	0.74	0.72	0.76
Overall SVI 2nd quartile	1.20	1.17	1.22
Overall SVI 3rd quartile	1.28	1.24	1.32
Overall SVI 4th quartile	1.42	1.36	1.47
year 2010 vs 2019	0.01	0.00	0.01
year 2011 vs 2019	0.10	0.10	0.10
year 2012 vs 2019	0.09	0.09	0.10
year 2013 vs 2019	0.16	0.16	0.16
year 2014 vs 2019	0.25	0.25	0.26
year 2015 vs 2019	0.32	0.32	0.33
year 2016 vs 2019	0.45	0.44	0.46
year 2017 vs 2019	0.59	0.57	0.60
year 2018 vs 2019	0.80	0.78	0.82

,

© 2024 Reynolds KR et al. *JAMA Network Open.*

d) Fully Adjusted

Effect	Odds Ratio	95% Confidence Interval	
Black	0.77	0.75	0.79
Hispanic	0.87	0.85	0.89
Other Race	1.06	1.03	1.09
Age	1.01	1.01	1.01
Female	0.98	0.97	0.99
Socioeconomic Status SVI 2nd quartile	1.10	1.08	1.13
Socioeconomic Status SVI 3rd quartile	1.09	1.06	1.12
Socioeconomic Status SVI 4th quartile	1.24	1.21	1.28
Household Composition /Disability SVI 2nd quartile	0.89	0.88	0.91
Household Composition /Disability SVI 3rd quartile	0.84	0.82	0.85
Household Composition /Disability SVI 4th quartile	0.77	0.75	0.79
Minority Status/Language SVI 2nd quartile	1.24	1.23	1.26
Minority Status/Language SVI 3rd quartile	1.32	1.29	1.34
Minority Status/Language SVI 4th quartile	1.65	1.62	1.69
Housing Type / Transportation SVI 2nd quartile	0.86	0.85	0.87
Housing Type / Transportation SVI 3rd quartile	0.76	0.75	0.78
Housing Type / Transportation SVI 4th quartile	0.74	0.73	0.76
Overall SVI 2nd quartile	1.19	1.17	1.22
Overall SVI 3rd quartile	1.28	1.24	1.32
Overall SVI 4th quartile	1.41	1.35	1.47
ACE	1.06	1.05	1.08

© 2024 Reynolds KR et al. *JAMA Network Open.*

Effect		Odds Ratio	95% Confidence Interval	
ARB		1.22	1.20	1.24
BB		1.16	1.15	1.17
CCB		0.94	0.93	0.95
NDHPCCB		1.31	1.29	1.33
Antiarrhythmic		1.60	1.57	1.62
Anti-platelet		0.91	0.90	0.93
CKD		0.64	0.63	0.65
COPD		0.89	0.88	0.90
Anti-diabetic		0.92	0.91	0.93
Heart Failure		0.81	0.80	0.82
Hypertension		0.94	0.92	0.96
Loop Diuretics		1.22	1.20	1.24
MI		0.64	0.63	0.65
Statin		1.23	1.21	1.24
Stroke		0.88	0.87	0.89
Thiazide Diuretics		0.98	0.97	1.00
year	2010 vs 2019	0.00	0.00	0.00
year	2011 vs 2019	0.09	0.09	0.09
year	2012 vs 2019	0.08	0.08	0.09
year	2013 vs 2019	0.14	0.14	0.15
year	2014 vs 2019	0.23	0.23	0.24
year	2015 vs 2019	0.30	0.30	0.31
year	2016 vs 2019	0.43	0.42	0.44
year	2017 vs 2019	0.56	0.55	0.58
year	2018 vs 2019	0.79	0.77	0.81

© 2024 Reynolds KR et al. *JAMA Network Open.*

eTable 4. Fully Adjusted Multinomial Regression Models Examining the Association Between Race/Ethnic Groups and DOAC Initiation Among Medicare Participants 2010-2019

Effect	Apixaban			Dabigatran			Rivaroxaban		
	Odds Ratio	95% Confidence Interval		Odds Ratio	95% Confidence Interval		Odds Ratio	95% Confidence Interval	
Black	0.82	0.80	0.84	0.70	0.68	0.73	0.83	0.81	0.85
Hispanic	0.83	0.81	0.86	0.88	0.85	0.92	0.92	0.89	0.95
Other Race	1.07	1.04	1.11	1.12	1.08	1.17	1.07	1.04	1.11

© 2024 Reynolds KR et al. *JAMA Network Open.*

eTable 5. Fully Adjusted Multinomial Regression Models Examining the Association Between Race/Ethnic Groups and DOAC Initiation Among Medicare Participants Who Are New Anticoagulant Users 2010-2019

Effect	Apixaban			Dabigatran			Rivaroxaban		
	Odds Ratio	95% Confidence Interval		Odds Ratio	95% Confidence Interval		Odds Ratio	95% Confidence Interval	
Black	0.76	0.73	0.78	0.64	0.61	0.68	0.73	0.71	0.76
Hispanic	0.77	0.74	0.79	0.81	0.77	0.85	0.83	0.80	0.86
Other Race	0.97	0.94	1.01	1.05	0.99	1.10	0.99	0.96	1.03

© 2024 Reynolds KR et al. *JAMA Network Open.*

eTable 6. Odds Ratios for Initiation of DOACS When Comparing Black and Hispanic Patients With White Patients From 2010-2019

a) Crude

Year	Hispanic			Black		
	Odds Ratio	95% Confidence Interval		Odds Ratio	95% Confidence Interval	
		Lower	Upper		Lower	Upper
2010	0.92	0.76	1.12	0.54	0.50	0.57
2011	0.89	0.83	0.95	0.54	0.50	0.58
2012	1.00	0.94	1.07	0.58	0.55	0.63
2013	1.02	0.95	1.09	0.69	0.65	0.74
2014	1.01	0.95	1.09	0.68	0.64	0.72
2015	0.99	0.92	1.07	0.75	0.71	0.80
2016	1.04	0.96	1.12	0.86	0.81	0.92
2017	0.97	0.89	1.05	0.83	0.78	0.89
2018	1.14	1.04	1.24	0.94	0.87	1.01
2019	1.30	1.17	1.45	1.08	0.99	1.18

© 2024 Reynolds KR et al. *JAMA Network Open.*

b) Fully Adjusted

Year	Hispanic			Black		
		95% Confidence Interval			95% Confidence Interval	
	Odds Ratio	Lower	Upper	Odds Ratio	Lower	Upper
2010	0.76	0.62	0.94	0.52	0.41	0.66
2011	0.77	0.71	0.82	0.59	0.55	0.63
2012	0.83	0.77	0.90	0.61	0.57	0.65
2013	0.90	0.84	0.97	0.75	0.71	0.81
2014	0.90	0.83	0.96	0.72	0.67	0.76
2015	0.89	0.82	0.96	0.79	0.74	0.84
2016	0.92	0.85	1.00	0.90	0.85	0.97
2017	0.82	0.75	0.89	0.85	0.79	0.91
2018	0.94	0.86	1.04	0.94	0.88	1.02
2019	1.00	0.89	1.12	1.03	0.94	1.13

Model adjusted for age; sex; race; Comorbid Conditions: CKD, Stroke, MI, Heart Failure, Hypertension, COPD, Baseline Medication Usage: Anti-platelet, Anti-diabetic, ARB, ACEi, Beta Blockers, Calcium Channel Blockers, NDHPCCB, Thiazide Diuretics, Loop Diuretics, Antiarrhythmic, Statins; Social Vulnerability Index Themes: Socioeconomic Status, Household Composition /Disability, Minority Status/Language, Housing Type/Transportation, Overall

© 2024 Reynolds KR et al. *JAMA Network Open.*

eFigure 2. Warfarin Uptake US Patients in the Lowest and Highest Quartiles of the Overall SVI Theme by Racial and Ethnic Group From 2010 to 2019

a) White

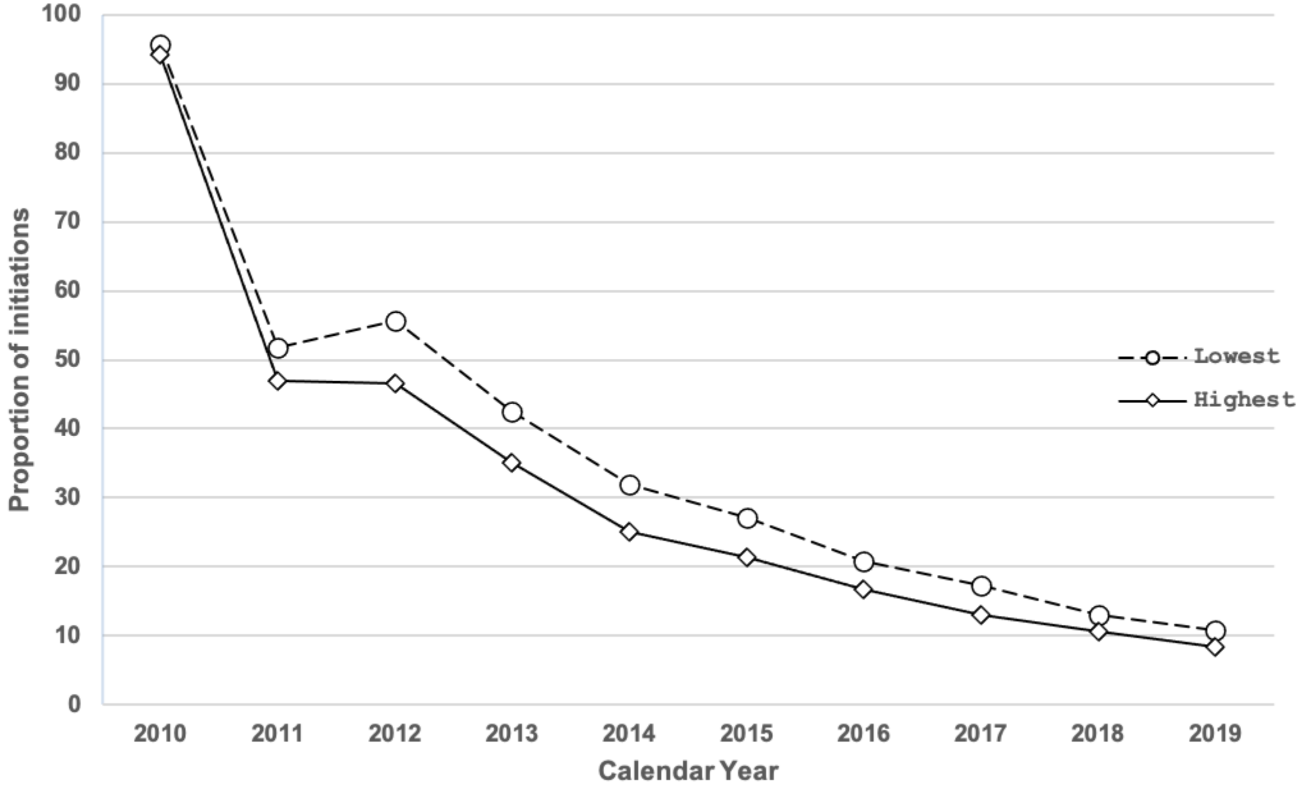

© 2024 Reynolds KR et al. *JAMA Network Open.*

b) Black

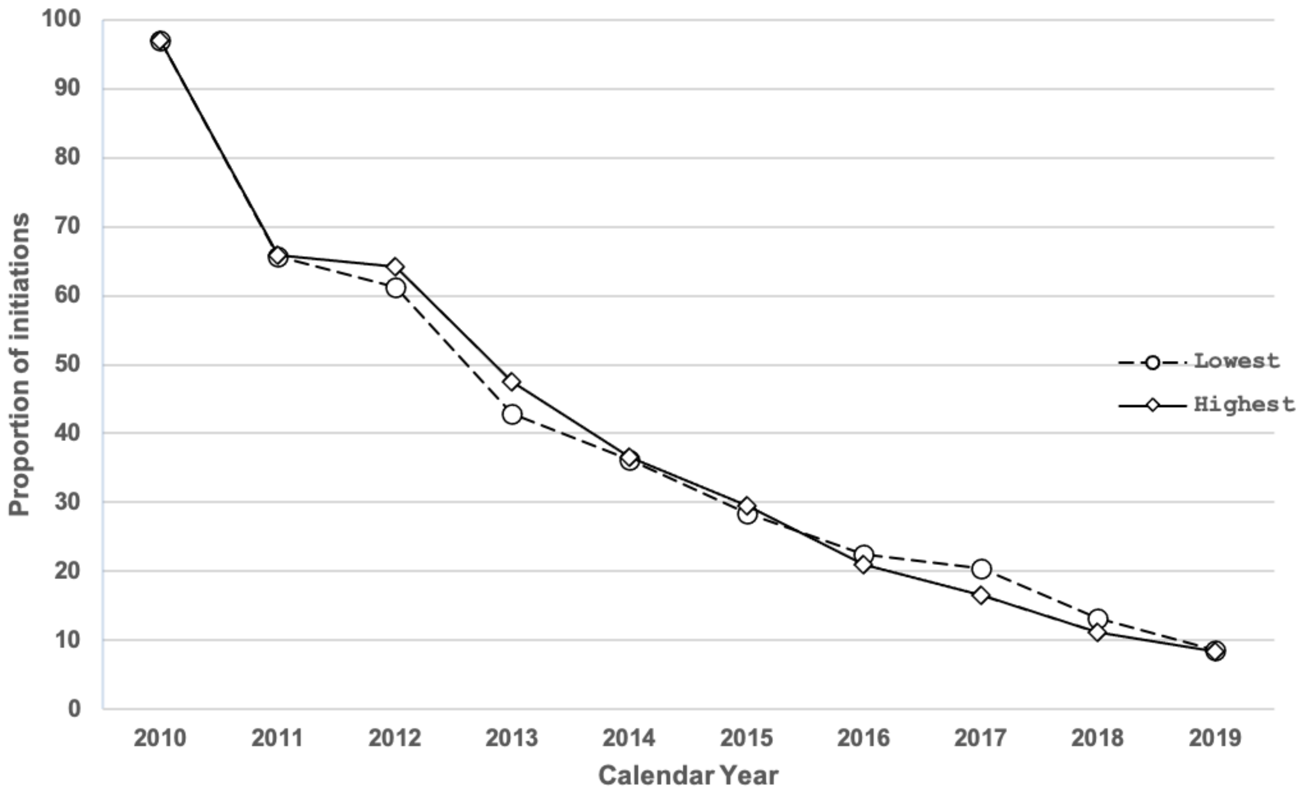

© 2024 Reynolds KR et al. *JAMA Network Open.*

c) Hispanic

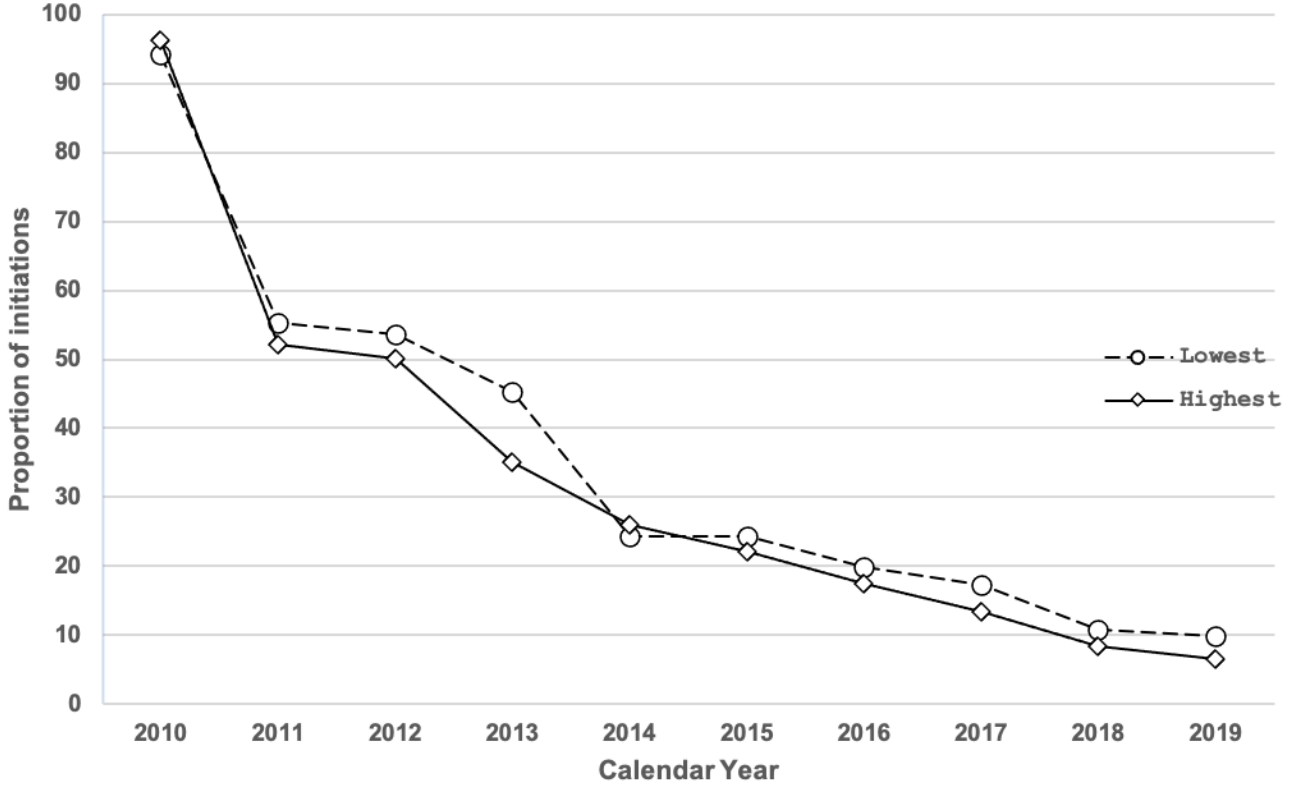

Overall SVI theme ranks each US county on 15 social factors across 4 themes: 1) socioeconomic status (below 150% poverty, unemployed, housing cost burden, no high school diploma, no health insurance); 2) household characteristics (aged 65 or older, aged 17 or younger, civilian with a disability, single-parent households, English language proficiency); 3) Racial and ethnic minority status (Hispanic or Latino (of any race); Black and African American, Not Hispanic or Latino; American Indian and Alaska Native, Not Hispanic or Latino; Asian, Not Hispanic or Latino; Native Hawaiian and Other Pacific Islander, Not Hispanic or Latino; Two or More Races, Not Hispanic or Latino; Other Races, Not Hispanic or Latino); and 4) Housing type & transportation (multi-unit structures, mobile homes, crowding, no vehicle, group quarters). Higher SVI ranking values indicating greater vulnerability, the lowest quartile includes the least vulnerable and the highest quartile includes the most vulnerable.

© 2024 Reynolds KR et al. *JAMA Network Open.*

eFigure 3. Proportion of DOAC Initiations Among White, Black, and Hispanic US Atrial Fibrillation Patients From 2010 to 2019 by CHA_2DS_2-VASc Score

a) CHA_2DS_2-VASc < Median

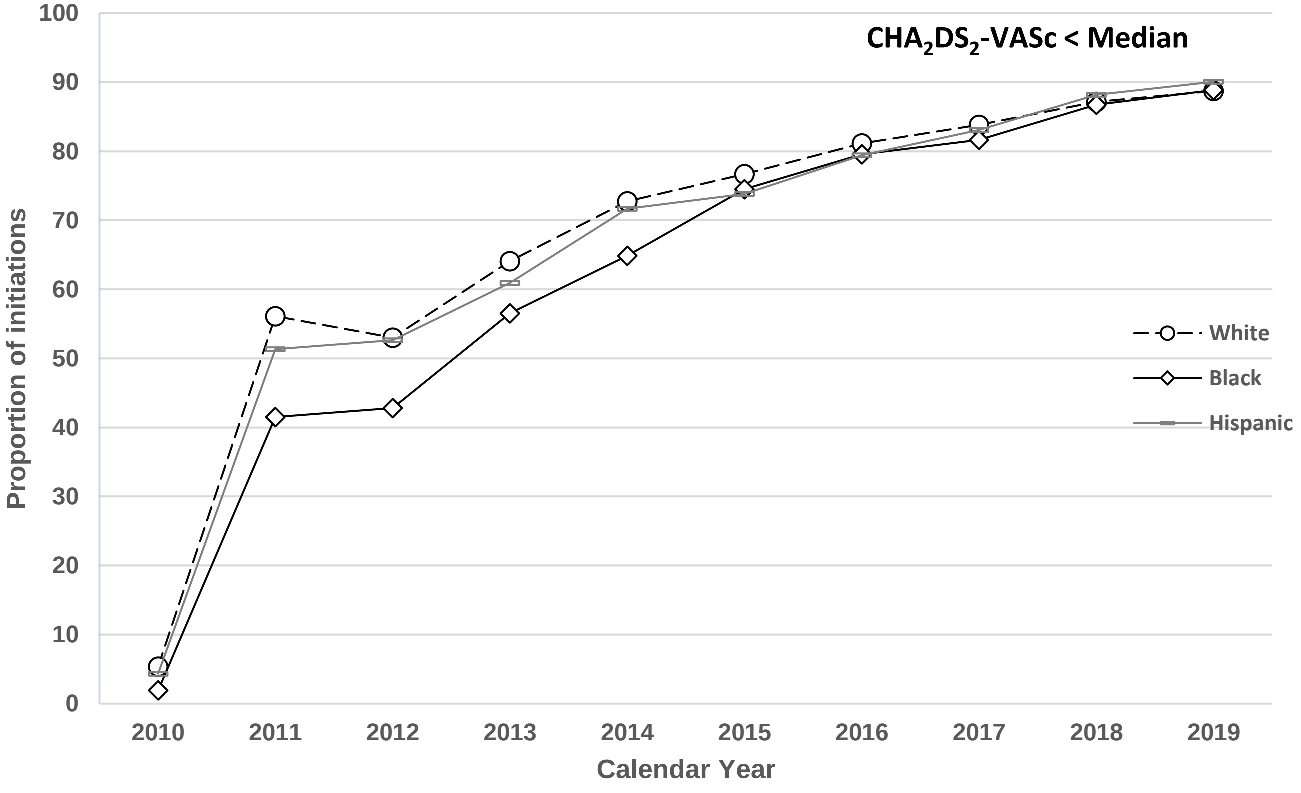

© 2024 Reynolds KR et al. *JAMA Network Open.*

b) CHA_2DS_2-VASc ≥ Median

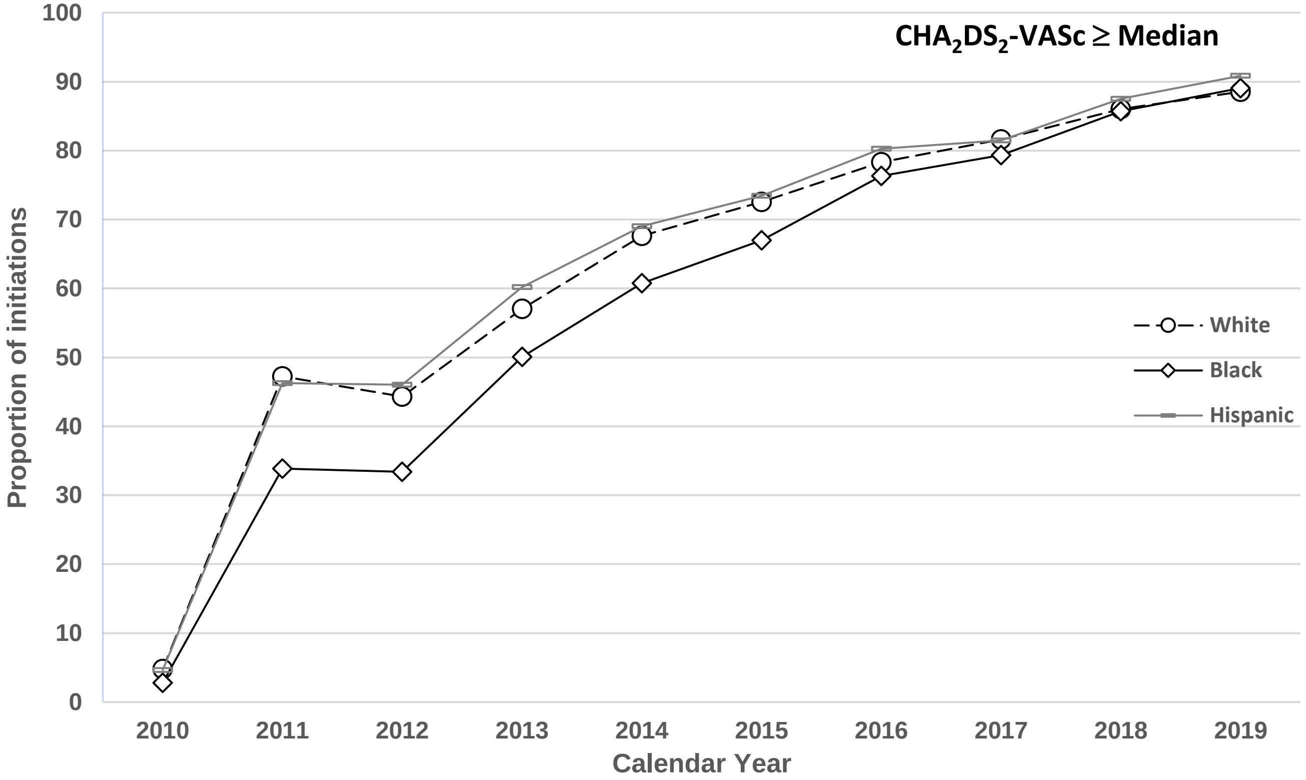

© 2024 Reynolds KR et al. *JAMA Network Open.*

c) CHA_2DS_2-VASc ≥ 75th Percentile

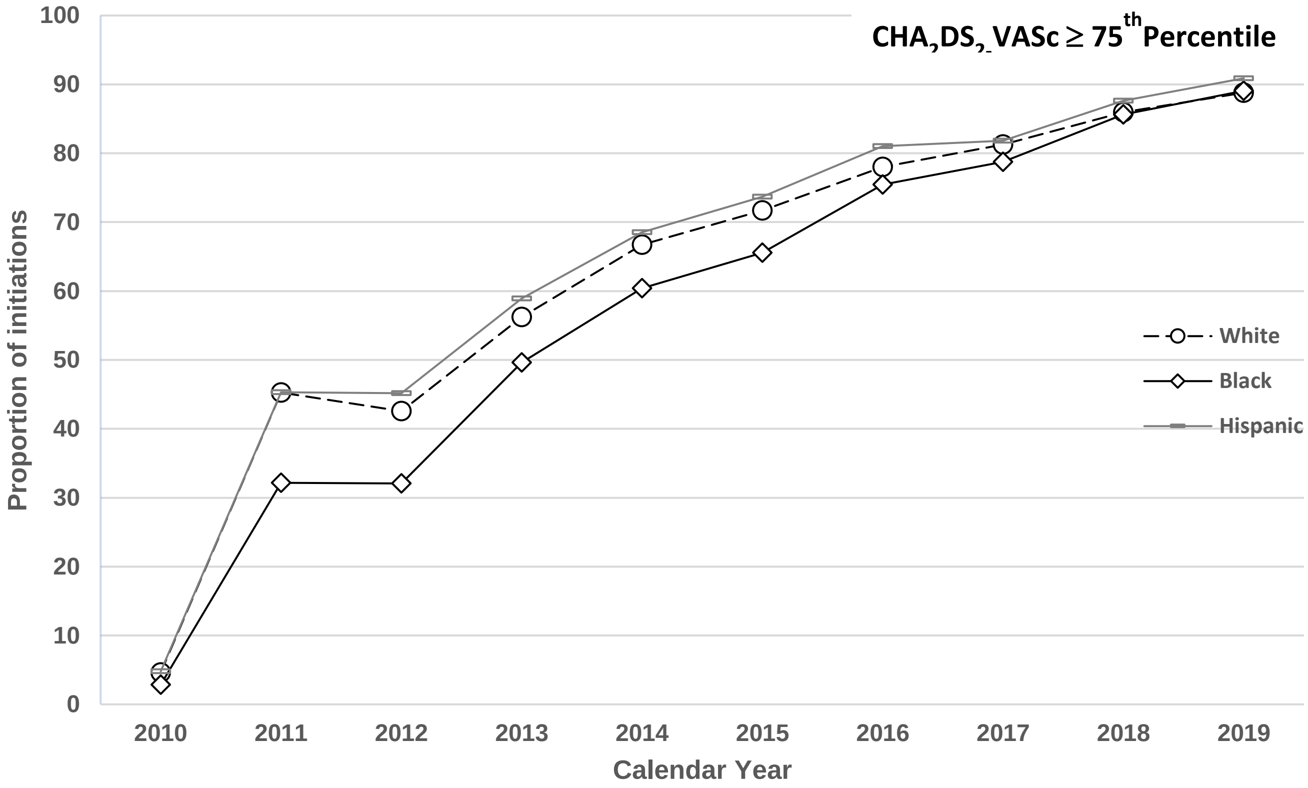

The median and 75th percentile CHA_2DS_2-VASc values for the overall population are 5 and 6 respectively.

© 2024 Reynolds KR et al. *JAMA Network Open.*
