# Supplementary material for: Racial and Ethnic Disparities in Initiation of Direct Oral Anticoagulants Among Medicare Beneficiaries
Source: JAMA Netw Open. 2024 May 6;7(5):e249465. doi: 10.1001/jamanetworkopen.2024.9465 (PMC11074810; doi:10.1001/jamanetworkopen.2024.9465)
Supplement: Supplement 2. — Data Sharing Statement [file jamanetwopen-e249465-s002.pdf]

Data Sharing Statement

Reynolds. Racial and Ethnic Disparities in Initiation of Direct Oral Anticoagulants Among Medicare Beneficiaries. *JAMA Netw Open*. Published May 06, 2024.
doi:10.1001/jamanetworkopen.2024.9465

Data
Data available: No
